# Cervical Cancer Awareness and Practice of Pap Smear Test Among Women with Gynecological problems

**Published:** 2018-06-30

**Authors:** Meena Thapa

**Affiliations:** 1 Department of Obstetrics and Gynaecology, Kathmandu Medical College, Sinamangal, Kathmandu, Nepal

**Keywords:** *awareness*, *cervical cancer*, *Pap smear test*

## Abstract

**Introduction:**

Cervical cancer is the most common cancer in women in Nepal. Pap smear test is the most important screening test for cervical cancer, which helps in reducing mortality from it. This study is to assess the knowledge of cancer cervix and practice of Pap smear test and to analyze the impact of educational status on them.

**Methods:**

This was a descriptive cross-sectional study, carried out among the married women who attended the Out Patient Department for gynecological problems at Kathmandu Medical College. Structured questionnaires were used to collect the data. The questionnaire consisted of three sections, first section included the demographic profile, second part included assessment of the knowledge of cancer cervix, and third part included evaluation of the utilization of Pap smear test.

**Results:**

A total of 205 married women were included in the study. Out of them,152 (74%) were aware of cancer cervix. Only 80 (39%) of women were aware of Pap smear test. Pap smear test coverage was 34 (16.6%) in studied population. Main reason of not doing Pap smear test was lack of knowledge of the test. High educational status of the women had significant positive impact on knowledge of cancer cervix and practice of Pap smear test.

**Conclusions:**

The knowledge of cancer cervix was good in our women, but knowledge and the practice of Pap smear test was poor. Good educational status of the women was found to influence the on knowledge of cancer cervix and uptake of Pap smear test.

## INTRODUCTION

Cervical cancer is the commonest cancer among women in our country with the incidence of 18.9%.^1^ It is the fourth common cancer in women worldwide^2^ and the second commonest cancer in Asia and Africa.^3^ Early marriage, multiple sex partners, smoking, and genital infection due to Human Papilloma Virus (HPV) are some important risk factors for it. It is reported that 80% of cancer cervix in Nepal, is attributed to high risk HPV infection.^4^

Mortality due to cancer cervix has decreased over the years in developed countries due to organized population based screening.^5^ In developing countries like Nepal, lack of awareness, cost of the test and unavailability of manpower are hindrances for routine screening of cervical cancer.^6^

Therefore, this study aimed to assess the knowledge of cancer cervix, to evaluate the practice of Pap smear test and impact of education status on knowledge of cancer cervix and practice of Pap test.

## METHODS

This was a cross sectional study, carried out in the women with gynaecological problems attending outpatient department (OPD) of Obstetrics and Gynaecology, Kathmandu Medical College. This study was carried out over a period of three months, from February 2014 to April 2014. The ethical approval was taken from Institutional Review committee of Kathmandu Medical College before initiation of the study. Married women who attended the OPD for gynecological problems on Mondays and Thursdays were included in the study. Written consent was taken from all the respondents for enrollment in the study. A structured, close ended questionnaire was used in the study, except for the demographic profile. The questionnaire had three sections. The first part consisted of eight questions, which were related to demographic profile of the women. The second part consisted of nine questions, which evaluated the knowledge of the cancer cervix and third part consisted of four questions evaluated the uptake of Pap smear test. Those women, who were aware of cervical cancer, were asked about the risk factors, symptoms, preventive measures and knowledge regarding prognosis of disease if treated in earlier stage. The second section consisted of questions with multiple response answers so women could choose more than one answers for a question. The practice of Pap smear test was asked to all women irrespective of their knowledge on cervical cancer. The data was recorded in Microsoft Excel sheet, and was analyzed using Statistical Package for Social Sciences version 20. Chi square Test was used to test the level of significance.

## RESULTS

This study included 205 married women. The mean age of women was 30.1±9.2 years. The mean age at marriage was 20.73±3.96 years. The mean number of births was 1.46. Total of 96 (46.8%) of women were educated till secondary level. Only 17 (8.3%) of them were illiterate.

Out of the 205 women, 152 (74.1%) were aware of cancer cervix. Among them, 130 (85%) women were aware of at least one risk factor of cervical cancer. Likewise, 112 (74%) women were aware of at least one symptom and nearly 98 (65%) of them knew about at least one preventive measure. Only two- fifths of the women were aware of Pap smear test whereas, more than three-fourths of women knew that cervical cancer can be cured if treated in early stage (Table 1).

**Table 1.** Knowledge of cancer cervix.

Variables	n (%)
Knows at least one risk factor of cancer cervix	130 (85.5)
Knows at least one symptom of cervical cancer	112 (73.7)
Knows at least one measure of prevention	98 (64.5)
Heard about Pap smear Test	60 (39.5)
Knows about cancer cervix can be treated in early stage	118 (77.6)

Out of the women who had heard of cancer cervix, nearly three fourth of them knew that genital tract infection as its main risk factor. Other factors like, poor genital hygiene, multiple sex partners and early sexual activities were also considered as important risk factors of cancer cervix. Only one third of women were aware that HPV infection is one of the risk factor of it. Nearly two third of women who had heard of cancer cervix, knew that abnormal per vaginal bleeding is one of the symptoms of cancer cervix. About 80 (54%) women among who had heard of cancer cervix were aware that avoidance of multiple sex partners as an important preventive measure. Only 26 (17%) women knew that HPV vaccination as a preventive measure of cancer cervix. It was found that media was main source of information about cancer cervix and Pap smear test (Table 2).

**Table 2.** Knowledge of risk factors, symptoms, preventive measures and sources of knowledge of cervical cancer among the women who heard of it.

	n (%)
Knowledge of risk factors	
HPV infection	46 (30.5)
Prolong genital tract	110 (72.8)
Infection	
Smoking	60 (39.7)
Multiple sex partners	93 (61.6)
Early pregnancy	84 (55.5)
Multiple pregnancy	91 (60.2)
Poor Genital Hygiene	99 (65.6)
Prolong use of OCP	65 (43)
Early sex and Marriage	85 (56.3)
Symptoms of cervical cancer	
Abnormal Vaginal Bleeding	88 (58)
Foul smelling Vaginal	76 (50)
Discharge	
Bleeding after intercourse	29 (19.2)
Preventive measures	
Avoidance of early sex/marriage	60 (39.7)
Avoidance of multiple sex partners	81 (53.6)
Avoidance of smoking	32 (21.2)
HPV vaccination	26 (17.2)
Sources of knowledge	
News/ Media	103 (67.7)
Health worker	52 (34.2)
Family/ friends	41 (26.9)
Women leaders	1 (0.6)

Out of the total women only 34 (16.6%) of them underwent Pap smear test in their lives. Lack of knowledge of the test was found to be the commonest reason of poor practice of this test (Table 3).

**Table 3.** Reasons of not undergoing Pap smear Test.

Reasons	n (%)
Lack of knowledge	133 (77.7)
Feeling healthy	15 (8.7)
Due to shyness	3 (1.7)
Thinking of painful procedure	8 (4.6)
Afraid of revealing cancer	2 (1.2)
Considering expensive	6 (3.5)
No exact reason	2 (1.2)

It was found that high educational status of the women had significant positive impact on knowledge of cancer cervix and practice of Pap smear test (Table 4).

**Table 4.** Relationship between Educational Status of women with awareness of cancer cervix and practice of Pap smear test.

Education status	Total number of women n (%)	Heard of cancer cervix		Had done Pap smear Test	
		Yes n (%)	No n (%)	Yes n (%)	No n (%)
**Illiterate**	**17 (8.3)**	**9 (52.9)**	**8 (47.1)**	**2 (11.8)**	**15 (88.2)**
**Literate**	**84 (40.9)**	**58 (69)**	**26 (31)**	**9 (10.7)**	**75 (89.3)**
**Primary level**	**8 (3.9)**	**4 (50)**	**4 (50)**	**4 (50)**	**4 (50)**
**Secondary level**	**53 (25.8)**	**40 (75.5)**	**13 (24.5)**	**11 (21)**	**42 (79)**
**Bachelor and above**	**43 (20.9)**	**41 (95.3)**	**2 (4.7)**	**8 (19)**	**35 (81)**
**Total**	**205 (100)**	**152 (74.1)**	**53 (25.9)**	**34 (16.6)**	**171 (83.4)**
**P value**		**0.001**		**0.047**	

## DISCUSSION

In our study, about 74% of women had heard of cancer cervix. The knowledge regarding risk factors and warning symptoms of cancer cervix was found to be good in our study. More than 50% of women knew about symptoms and its risk factors, but less than 50% of them were aware of preventive measures. Knowledge and practice of Pap test was found to be low however, it was higher than the national record. High educational status of the women was found to have a positive impact in the knowledge of cancer cervix and practice of Pap smear test.

A study from Dharan had reported that 90% of the educated women were aware of cancer cervix with adequate knowledge in 47% of them. ^7^ A study from Udayapur district had reported that adequate knowledge of cancer cervix was found in 63.3% of women.^8^ Similar to our study, 77% of women from Rukum had knowledge of cancer cervix. They also found that 71% of them were aware of its warning signs/symptoms.^9^ On the other hand, a hospital based study from India had reported a low level of knowledge regarding cancer cervix. ^10^ They found that less than one third of women had knowledge regarding the risk factors and symptoms of cancer cervix. Knowledge regarding symptoms of cancer cervix was found relatively better among the urban women from Nigeria^11^ whereas women from Ethiopia had only 46.3% of comprehensive knowledge of cancer cervix.^12^ A very poor knowledge of cancer cervix was found in Ghana. They reported that 68.4% of women had never heard of this cancer and more than 90% of them did not have any knowledge on its risk factors,^13^ it may be due to our women were better educated than them. Similar to our respondents, Nigerian women also considered that abnormal vaginal bleeding was an important symptom of cancer cervix^11^ and they also considered media as the important source of information. Likewise, most of the women from Rukum got information of cancer cervix from television.^9^

The cervical cancer screening coverage is only 2.8% in our country.^4^ The main reason behind not undertaking Pap smear test in our women was lack of knowledge regarding the test. Similar reason was reported by the study done in a hospital from India.^13^ We found that our pap smear test coverage is higher than the national data. The studies from Udayapur^8^ and Dharan^7^ also had reported a less coverage of Pap smear test. The test coverage was found much higher in a population based study done in Bharatpur municipality.^14^ One of the leading cancer center is located in Bharatpur. The higher rate of test coverage in Bharatpur area may be due to easy availability of the test. Low level of Pap smear test coverage was found in the hospital based study from India.^10^ They reported that only 9.5% of educated women underwent Pap smear test. Pap smear test utilization was found to be poor not only among our women but also in most of the developing countries of Africa. They reported that pap smear test coverage of 0.8 to 15.4%.^11,13^

A study from Ghana also reported that important barriers of the cervical cancer screening were lack of screening sites and poor knowledge of the test.^13^ As in our study, other studies also reported that high educational status of the women had a significant impact on the knowledge, attitude and practice of the Pap smear test.^7,10^

## CONCLUSIONS

The knowledge of cervical cancer was good in our women, but knowledge of Pap smear test and uptake of this test was poor. Educational status of the women was found to have positive impact on the knowledge of cancer cervix and uptake of the Pap smear test.
